# Effect of PBAT-g-MAH Compatibilization and Bamboo Flour Loadings on Melt Flow and Early Soil–Compost Mineralization of PLA Biocomposites for FFF 3D Printing

**DOI:** 10.3390/polym17243240

**Published:** 2025-12-05

**Authors:** César A. Paltán, Jorge I. Fajardo, Diana V. Rodriguez, Edwuin Carrasquero

**Affiliations:** 1 New Materials and Transformation Processes Research Group (GiMaT), Universidad Politécnica Salesiana, Cuenca 010105, Azuay Province, Ecuador; cpaltan@ups.edu.ec (C.A.P.); drodriguezp@ups.edu.ec (D.V.R.); 2 Universidad Estatal de Milagro, Milagro 091050, Provincia del Guayas, Ecuador; ecarrasqueror@unemi.edu.ec

**Keywords:** PLA, bamboo fiber, compatibilizer, PBAT-g-MAH, melt-flow index, ASTM D5988, mineralization, FFF/FDM

## Abstract

Objective. To determine how bamboo loadings (2.5–5 wt%) and compatibilization with PBAT-g-MAH (BP-1, 10 wt%) affect melt flow and early-time mineralization of PLA biocomposites under near-ambient soil–compost conditions (ASTM D5988), while using PBAT-g-GMA (BP-2) only as a melt-flow screening reference. Methods. Melt flow index (MFI, ASTM D1238, 2.16 kg; 190/210/230 °C) was first measured for neat PLA and PLA/BP-1/BP-2 blends to select a printable matrix. PLA/10BP-1 composites containing 2.5–5 wt% bamboo were then compounded, extruded as bars for biodegradation tests, and validated by FFF printing. Biodegradation was quantified from titrimetric CO_2_ evolution in soil–compost reactors at 21 ± 2 °C and pH ≈ 7 (triplicate specimens plus triplicate blanks; mean ± SD and endpoint statistics). ATR-FTIR was used to support mechanistic interpretation. Results. BP-1 markedly increased MFI relative to neat PLA, whereas BP-2 remained close to the neat matrix, consistent with epoxy-driven coupling that can raise viscosity. Under ambient burial, all materials exhibited very low mineralization over 0–23 days; PLA/10BP-1/2.5B and PLA/10BP-1/5B showed a slight increase in net CO_2_ evolution compared with neat PLA, but the differences remained modest and within the experimental uncertainty, reflecting a balance between bamboo’s pro-hydrolytic effect and the sealing action of PBAT-g-MAH compatibilization. Significance. The data delineate a printing–degradation window in which PLA/10BP-1 with 2.5–5 wt% bamboo combines easy processing and short-term durability while preserving industrial compostability at end-of-life.

## 1. Introduction

The accelerating accumulation of plastic waste has intensified the search for bio-based, end-of-life-manageable polymers suitable for high-volume applications. Polylactic acid (PLA) has emerged as the leading commodity biopolyester, owing to its renewable origin and favorable stiffness/strength balance, yet its environmental performance depends strongly on *how* and *where* it is disposed and on the material’s formulation and processing history. Under hydrolytic and microbial action, PLA degrades primarily through ester scission followed by bioassimilation of soluble oligomers; rates increase with temperature (particularly above the glass-transition), humidity and the presence of specific microbial consortia and enzymes [1,2,3,4,5,6].

Natural fibers are an attractive route to reduce petrocontent and tune properties of bioplastics. Among them, bamboo, a fast-growing lignocellulosic resource, has been widely studied as a reinforcement/filler in polyesters. Compatibilization (e.g., MAH-based) can strengthen interfacial adhesion, reduce interfacial water uptake, and thereby modulate both mechanical performance and moisture-assisted degradation pathways [7,8,9,10].

In PLA systems, *water-induced interfacial decohesion* has been evidenced in miscanthus-reinforced PLA, underscoring the role of fiber–matrix interfaces in wet environments [9].

Concurrently, material-extrusion (FFF/FDM) 3D printing has become a dominant shaping route for PLA and PLA-based composites. Processing parameters such as nozzle temperature, layer thickness, raster and infill architecture govern interlayer bonding, porosity/microvoids and crystallinity, which in turn dictate both mechanical durability and the exposure pathways for hydrolysis and microbial colonization [11,12,13,14,15,16,17,18].

For fiber-filled PLA, increasing infill density and optimizing patterns tends to reduce percolative void networks and retard water ingress; sub-optimal settings can accelerate it [16,17,18,19,20,21,22,23].

Here we address two practical gaps: (i) quantitative evidence for early-time mineralization (≤ 3–4 weeks) of extrusion-processed or printed PLA biocomposites at ~21 °C in soil–compost mixtures, and (ii) the role of low fiber loadings under deliberate compatibilization in moderating water pathways and hydrolysis. Therefore, the main objective of this study is to (i) select a printable matrix via MFI screening (neat PLAs vs. PBAT-g-MAH/GMA) and (ii) quantify how 2.5–5 wt% bamboo in PLA/10BP-1 affects early mineralization (ASTM D5988-18) under near-ambient soil–compost conditions, with supporting ATR-FTIR evidence for MAH-driven interfacial reactions.

The net effect of natural fibers on PLA biodegradation is system- and environment-dependent. Across soil, compost, aqueous and fungal assays, many studies report that fibers accelerate mass loss or molecular-weight reduction relative to neat PLA by increasing hydrophilicity, providing crack-nucleation sites and enabling biofilm attachment—whereas in specific systems, interfaces and temperatures, the effect can be neutral or retarding [12,13,24,25,26,27,28,29,30,31,32,33,34,35].

For instance, cellulose-based reinforcements and reed fibers have been shown to increase mass loss or accelerate property decay under compost/soil exposure, while some wood-based systems with compatibilizers or optimized printing exhibit moderated or even reduced degradation [34,36,37].

Beyond general trends, multiple controlled studies quantify how natural fibers can accelerate PLA disintegration under compost/soil exposure, while also revealing the strong dependence on fiber chemistry, surface treatments, loading, and environment. For example, kenaf–PLA composites reached 15.9–17.1% weight loss under composting versus ~4% for neat PLA, and flax–PLA showed 20–25% mass loss within 50–60 days in agricultural soil; in a matrix modified by acrylic-acid grafting (PLA-g-AA) with sisal reinforcement, complete degradation occurred within 6–10 weeks [38,39,40].

Mechanical durability under burial also decays faster in composites: a PLA/hemp system lost ~34% of strength in four months versus ~11% for neat PLA [41]. At the same time, kinetic outcomes hinge on the interplay between fiber nucleation and pro-hydrolytic effects: recent PLA/hemp work shows that fibers can simultaneously nucleate crystallinity (stiffening) and promote hydrolysis (softening), with the net trajectory governed by printing/processing and exposure conditions [42]. These quantified exemplars sharpen the rationale for our focus on low fiber loadings and intentional compatibilization, where improved interfacial cohesion and controlled porosity/crystallinity may dampen early-time mineralization relative to neat PLA, despite added hydrophilicity—precisely the regime evaluated here.

Despite this rich literature, two practical gaps persist for design-for-end-of-life of printed consumer parts. *First*, much quantitative evidence derives from elevated temperatures (e.g., industrial compost, enzymatic media) rather than ambient soil–compost environments where many items may ultimately reside; early-time mineralization (<1–2 months) at ≈20–25 °C for printed biocomposites remains sparsely quantified [25,26,43,44,45]. *Second*, there is limited mechanistic data at low fiber loadings in PLA systems intentionally compatibilized to favor processing (e.g., MAH-based agents), where improved interfacial cohesion could damp the water-uptake pathways by which fibers otherwise accelerate disintegration [7,10,46].

To address these gaps, we investigate early-stage mineralization (0–23 days) of 3D-printed PLA and PLA + bamboo composites under a soil–compost mixture near neutrality (pH ≈ 7) and ≈21 ± 2 °C, using ASTM D5988 titrimetric CO_2_ evolution. We intentionally employ low bamboo loadings (2.5 and 5 wt%) and a fixed compatibilizer level (10 wt% BP-1) selected from a prior melt-flow screening to ensure printable viscosity and consistent interlayer bonding. Literature supports two competing hypotheses in this regime: *(i)* bamboo (and plant fibers broadly) may increase hydrophilicity and microcrack pathways, accelerating hydrolysis and microbial access; *(ii)* compatibilization and dense printed infill may lower effective water diffusivity through improved interfacial sealing and crystallinity control, thereby retarding early mineralization relative to neat PLA [7,12,13,14,15,16,19,22,24,25,26,27,28,30,36,47,48].

Our key outcome is that very low mineralization at 23 days was observed for all formulations, with PLA/10BP-1/2.5B and PLA/10BP-1/5B exhibiting only slightly higher mineralization than neat PLA. This is consistent with a regime where bamboo’s pro-hydrolytic role is largely damped but not suppressed by compatibilization and dense morphology, rather than strongly amplified, and agrees with reports that fiber effects can be modest or even invert when interfacial bonding and processing minimize porosity or when temperatures remain below composting setpoints [15,16,17,19,22,25,49].

## 2. Materials and Methods

### 2.1. Materials and Composite Formulations

Polylactic acid (PLA) pellets (TOTALenergies, Courbevoie, France; pre-dried 80 °C/24 h) were used as the matrix. For melt-flow screening (ASTM D1238-21), several neat PLA grades were compared (e.g., Smartfil PLA, Luminy^®^ LX175, PLA 2003, Total Energize—names are given once for traceability); thereafter, we refer to them uniformly as neat PLA grades. Downstream, composite codes are standardized as PLA, PLA/10BP-1, PLA/10BP-1/2.5B, PLA/10BP-1/5B, and PLA/10BP-2 (BP-2 used only for MFI).

Two compatibilizers (COACE Chemical), PBAT-g-MAH (BP-1) and PBAT-g-GMA (BP-2), were evaluated as processing aids/coupling agents with PTE. Declared grafting level: “High (≥0.8 wt%)”. Storage: dry, dark. BP-1 is the study focus; BP-2 was used only for MFI screening.

The lignocellulosic reinforcement used was bamboo flour (Guadua angustifolia Kunth) sieved to <150 µm (passing #100) and pre-dried at 80 °C for 24 h, at concentrations of 2.5 and 5 wt%, while PBAT-g-MAH (BP-1, COACE Chemical) was incorporated at 10 wt% in each formulation as a coupling agent to improve compatibility between the polymer phase and the lignocellulosic phase.

**Figure 1:**
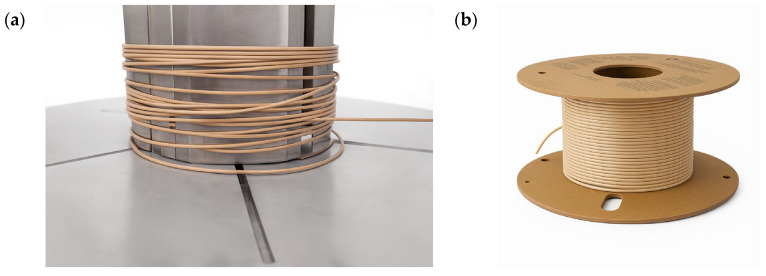
PLA/10BP-1/2.5B filament used as feedstock for FFF printing: (**a**) close-up of the as-extruded filament (Ø 1.75 ± 0.05 mm) winding around the take-up drum; (**b**) representative spool of the same filament after extrusion.

Batch-level compositions for biodegradation testing were prepared as 40 g mixes and then scaled to ~20 g specimen mass per reactor, and the two biocomposite formulations used in soil–compost tests were PLA/10BP-1/2.5B (PLA 35.1 g; bamboo 1.0 g; BP-1 3.9 g; total 40 g) and PLA/10BP-1/5B (PLA 34.6 g; bamboo 2.0 g; BP-1 3.4 g; total 40 g); for reference, a Smartfil PLA specimen (neat PLA) was tested in parallel under identical conditions. Dry blends were pre-mixed and compounded in a Brabender Plastograph 50EHT3Z/Reino (Brabender GmbH & Co. KG, Duisburg, Germany) at 170 °C, 40 rpm. For printing validation, we used filament extruded in aCollin Teach-Line E20T single-screw extruder (COLLIN Lab & Pilot Solutions GmbH, Maitenbeth, Germany) (draw control to target Ø 1.75 ± 0.05 mm). For biodegradation testing, extruded bars/pellets (not printed) were prepared; typical per-reactor specimen mass ≈ 20 g. A representative extruded filament obtained from the PLA/10BP-1/2.5B matrix is shown in Figure 1a,b.

### 2.2. Melt Flow Index (MFI) Tests

MFI was measured according to ASTM D1238 (2.16 kg) at 190, 210 and 230 °C. n = 10 per condition. Results are reported as mean ± SD. BP-2 was included solely in comparison with BP-1. For clarity, BP-2 (PBAT-g-GMA) is used exclusively for MFI determination; in all subsequent processing and biodegradation tests, BP-1 (PBAT-g-MAH) is used at 10 wt% in PLA. Specifically, MFI was measured for Smartfil PLA, neat PLA grades (PLA LX175, PLA 2003, PTE), PTE + BP-1 at 5/10/20 wt%, and PTE + BP-2 at 5/10/20 wt%, each at 190, 210 and 230 °C.

### 2.3. Degradation Analysis

**Figure 2:**
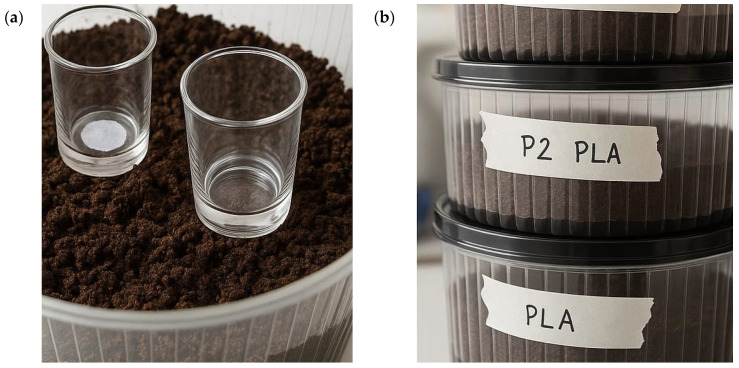
Soil–compost biodegradation setup used for ASTM D5988 tests: (**a**) glass sample holders embedded in the soil–compost matrix before sealing the reactor; (**b**) labeled containers with soil–compost and PLA-based formulations (e.g., PLA and PLA/10BP-1/bamboo) arranged as replicate CO_2_-capture reactors.

For CO_2_ evolution, we used bars/pellets to avoid confounding porosity/architecture effects from FFF and isolate composition/compatibilization. Biodegradation was assessed in soil–compost reactors (pH ≈ 7; 21 ± 2 °C; RH 50–70%) following ASTM D5988 with titrimetric CO_2_ capture (KOH) and HCl back-titration. Triplicate reactors (n = 3) were run per formulation, and three blanks were operated in parallel to monitor drift; blank solutions were periodically refreshed to maintain stability. The soil–compost biodegradation setup, including the CO_2_ capture reactors, KOH traps, and replicate configuration, is shown in Figure 2a,b.

CO_2_ was sampled every 3–4 days during the first three weeks and weekly thereafter. Net CO_2_ (Xₙ) was computed as the blank-corrected HCl volume; cumulative CO_2_ (mg) and % mineralization vs. theoretical carbon content (%C) were obtained by standard stoichiometry. Occasional negative Xₙ increments in low-signal regimes were treated as blank-drift artifacts and propagated with their uncertainty without truncation; interpretation relies on the cumulative signal, and its SD. Endpoint comparisons used one-way ANOVA + Tukey HSD (α = 0.05); non-parametric Kruskal–Wallis + Dunn–Šidák were used for confirmation.

Blank handling and negative increments. We operated triplicate blanks in parallel and refreshed capture solutions periodically. Small negative day-to-day Xₙ increments occasionally arose when signals approached the titrimetric detection limit; these were treated as blank-drift artifacts and propagated with their uncertainty without truncation, while interpretation relies on cumulative CO_2_ and SD bands.

The biodegradation media were prepared by controlling the pH with a 5:1 substrate/water ratio. A pH range of 6–8 was maintained to avoid conditions that could alter microbial activity or CO_2_ quantification. To quantify CO_2_ generated by biodegradation, each CO_2_ capture tank contained 20 mL of 0.5 N KOH and 50 mL of distilled water.

**Figure 3:**
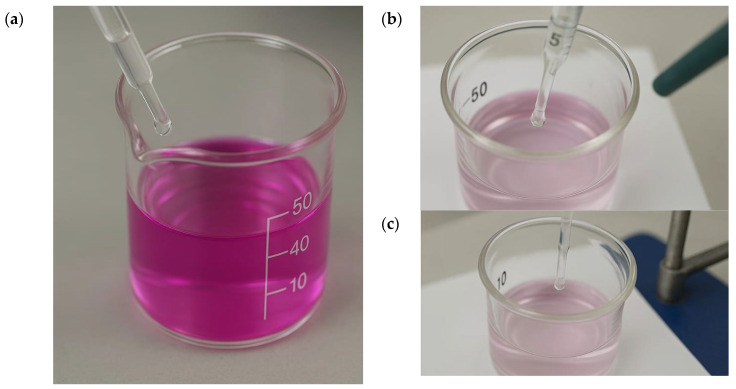
Standardization of the KOH solution used for CO_2_ capture by titration with 0.25 N HCl: (**a**) initial strongly pink KOH/phenolphthalein solution before titration; (**b**) approach to the endpoint as the color fades; (**c**) final colorless solution at the titration endpoint.

Before each titration, containers were weighed on an analytical balance; their contents were then homogenized, and liquids were removed or replenished as appropriate: the masses of the containers with KOH were measured before and after titration, and the 50 mL in the containers with distilled water were replaced with a fresh aliquot. The progressive color change from strongly pink to colorless at the endpoint is illustrated in Figure 3a–c.

To maintain constant soil moisture, corrections were made by adding distilled water when mass losses were detected; the standard make-up was 1.2 g per titration cycle. Finally, all experimental data (HCl volumes, initial and final masses, and moisture corrections) were compiled and used to calculate the CO_2_ generated and evaluate the biodegradation of the mixtures. Captured CO_2_ was quantified by titrating the KOH solution with standardized 0.25 N HCl using phenolphthalein as indicator, to the color change from pink to colorless.

### 2.4. Degradability Determination

The biodegradability of the polymers was quantified by measuring the carbon dioxide (CO_2_) released during incubation under controlled composting conditions. The procedure followed ISO 14855-1:2012 (Determination of the Ultimate Aerobic Biodegradability of Plastic Materials under Controlled Composting Conditions—Method by Analysis of Evolved Carbon Dioxide—Part 1: General Method. International Organization for Standardization: Geneva, Switzerland, 2012), which establishes a closed CO_2_ capture system with subsequent acid–base titration. During the experiment, the CO_2_ produced was absorbed in a potassium hydroxide solution (0.5 N KOH), reacting as

2 KOH + CO_2_→ K_2_CO_2_+ H_2_O (1)

KOH + HCl → KCl + H_2_O (2)

From stoichiometry, one mole of CO_2_ corresponds to two moles of HCl:

1 mol CO_2_ = 2 mol HCl (3)

#### 2.4.1. Net Amount of Carbon Dioxide Produced

The amount of CO_2_ captured was determined by titration with hydrochloric acid (0.25 N HCl), using phenolphthalein as the indicator. The net titrant volume attributable to the sample is

(4) $$\bar{X_{N}\;}=\bar{X_{B}}-\bar{X_{T}}$$

where

$\bar{X_{B}}$: average mL of HCl used to titrate the blank control

$\bar{X_{T}}$: average mL of HCl used to titrate the test sample (or positive control)

$\bar{X_{N}\;}$: net mL of HCl corresponding only to CO_2_ generated by the sample (or positive control)

#### 2.4.2. Amount of CO_2_ Generated by the Material

(5) $$g\;{\mathrm{CO}}_{2}=\;C\;\cdot \;\bar{X_{N}\;}\;\cdot \;\mathrm{MW}\;\cdot \;\left(\frac{1\;\mathrm{mol}\;{\mathrm{CO}}_{2}}{2\;\mathrm{mol}\;\mathrm{HCl}}\right)\cdot \;\left(\frac{1\;L}{1000\;\mathrm{mL}}\right)$$

where

C: HCl concentration = 0.25 M

MW: molecular weight of CO_2_ = 44 g·mol^−1^

#### 2.4.3. Theoretical CO_2_ Amount


(6) $${\left(g\;{\mathrm{CO}}_{2}\right)}_{theoretical}={\left(g\;C\right)}_{theoretical}\times \left(\frac{44}{12}\right)$$

using MW(CO_2_) = 44 g·mol^−1^ and MW(C) = 12 g·mol^−1^.

#### 2.4.4. Percentage of Carbon Dioxide Produced


(7) $$\%\;{\mathrm{CO}}_{2}=\left[\frac{g\;{\mathrm{CO}}_{2}}{{\left(g\;{\mathrm{CO}}_{2}\right)}_{theoretical}}\;\right]\times 100\%$$

This percentage is evaluated at each measurement and then accumulated over the test to obtain the total CO_2_ produced and, consequently, the extent of biodegradation of the material.

#### 2.4.5. Standard Deviation of the Percentage of Biodegradation

(8) $$S_{e}=\sqrt{\frac{S_{T}^{2}}{n_{T}}+\frac{S_{B}^{2}}{n_{B}}}\times \frac{1}{{\left(g\;C\right)}_{theoretical\;}}\;$$

where

$S_{T}$: standard deviation for the sample (or positive control)

$n_{T}$: number of replicates for the sample (or positive control)

$S_{B}$: standard deviation for the blank

$n_{B}$: number of replicates for the blank

${\left(g\;C\right)}_{theoretical\;}:$ theoretical mass of carbon

This calculation framework provides a quantitative basis for evaluating biodegradation. By combining measured HCl consumption with CO_2_ generation, the dynamics of the system can be characterized. The explicit stoichiometric relationships and statistical parameters ensure accuracy and reproducibility, enabling identification of degradation patterns and validation of material performance.

### 2.5. FTIR-ATR Characterization Methods

Chemical characterization was performed using attenuated total reflectance Fourier transform infrared spectroscopy (FTIR-ATR) with a Nicolet™ iS™ 10 spectrometer (Thermo Fisher Scientific Inc., Waltham, MA, USA) equipped with a Smart iTX accessory and a diamond crystal. The system employed a DTGS detector with a KBr window, a KBr beam splitter, and an internal infrared radiation source.

Spectral processing and analysis were performed using OMNIC™ software (Thermo Fisher Scientific Inc., Waltham, MA, USA), applying baseline correction and absorbance normalization. Each spectrum was recorded over 4000–600 cm^−1^ at 4 cm^−1^ resolution with 32 co-added scans. Samples analyzed were neat PLA, PLA/10BP-1 (PBAT-g-MAH, 10 wt%) and PLA/10BP-1/2.5B (bamboo flour 2.5 wt%). For each formulation, three independent spots were measured to ensure repeatability, applying constant contact pressure on the ATR crystal. Spectra were baseline-corrected and normalized at 1452–1455 cm^−1^ (CH bending of PLA) prior to comparison. Diagnostic bands tracked included PLA ν(C=O) of the ester at ~1745–1755 cm^−1^, C–O–C stretching at ~1180–1050 cm^−1^, CH_3_ stretching at ~2995–2945 cm^−1^, O–H stretching envelope at ~3600–3200 cm^−1^ (hydroxyls, moisture/lignocellulose) and MAH-related features in ~1850–1780 cm^−1^ (anhydride overtones/combination bands) in agreement with typical assignments reported for PLA-based and MAH-compatibilized systems [7,9,10,30,47,48].

### 2.6. Printing Validation

**Figure 4:**
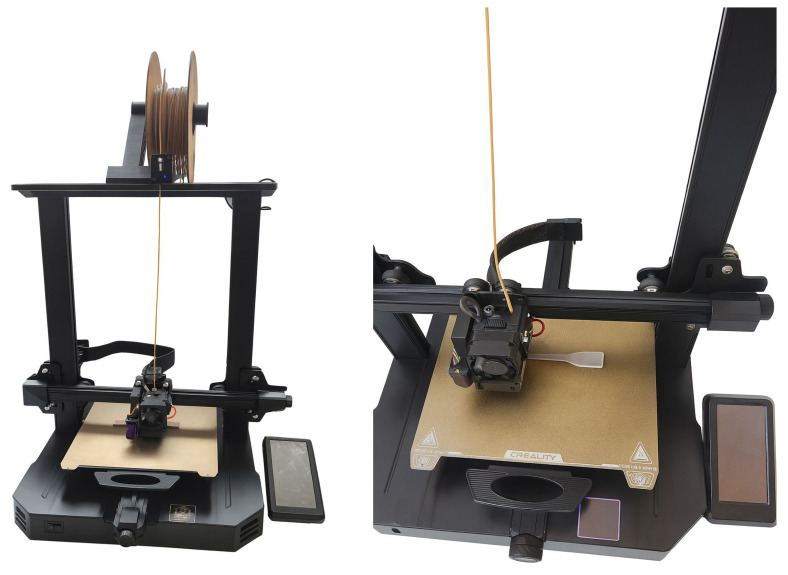
Representative FFF-printed specimens produced with PLA/10BP-1 with and without bamboo (2.5–5 wt%) under the selected processing parameters (Creality Ender-3 S1 Pro, 0.4 mm nozzle, 0.20 mm layer height, 100% rectilinear infill, two perimeters, ± 45° rasterization).

To evaluate the printability of the biocomposites, we performed an FFF test (Creality Ender-3 S1 Pro, 0.4 mm nozzle, 0.20 mm layer height, 100% rectilinear infill, two perimeters, ±45° rasterization). Representative printed specimens obtained under these conditions are shown in Figure 4. Visual inspection confirmed continuous bead formation, absence of gross interlayer voids, and comparable surface quality for PLA/10BP-1 with and without 2.5–5 wt% bamboo, indicating that low bamboo loadings do not compromise printability under the selected parameters.

### 2.7. Statistics and QA

Time-series are summarized as mean ± SD (n = 3). Because endpoint mineralization values remain in a sub-percent regime and n is small, formal hypothesis tests have limited power; we therefore restrict inference to exploratory one-way ANOVA (day 23 percent mineralization, α = 0.05) and a Kruskal–Wallis check to verify robustness. Effect-size metrics (partial η^2^, Hedges’ g) were used to gauge the practical relevance of any differences. Given these limitations, statistical outcomes are interpreted descriptively (trends rather than strict rankings) rather than as definitive evidence of large differences between formulations. QA checks include blank stability trending, reactor-to-reactor SD visualization, and mass-based moisture make-up using the container mass logs.

**Figure 5:**
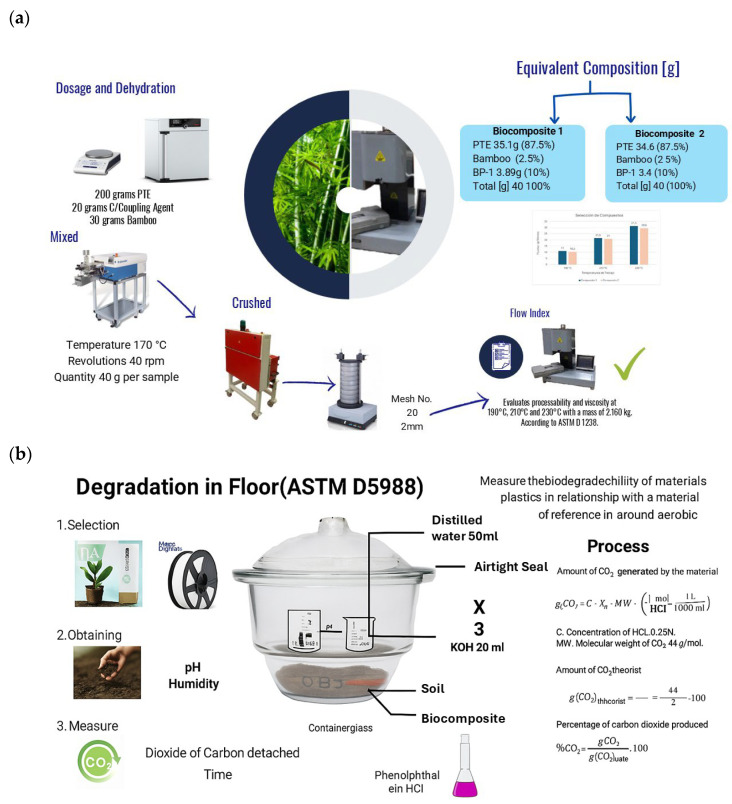
Process flow and biodegradation setup used in this study: (**a**) preparation and screening of PLA/10BP-1/bamboo biocomposites, including drying of PLA, bamboo flour, and PBAT-g-MAH (BP-1) at 80 °C for 24 h, Brabender Plastograph 50EHT3Z/Reino compounding at 170 °C and 40 rpm, crushing and sieving (mesh No. 20, 2 mm) to obtain granules, extrusion into bars for biodegradation tests (≈20 g per reactor) and optional filament (Ø 1.75 ± 0.05 mm) for FFF validation, followed by melt-flow index (MFI) measurements according to ASTM D1238; (**b**) schematic of the soil–compost biodegradation protocol (ASTM D5988), showing the soil–compost matrix, biocomposite specimen, distilled water and KOH CO_2_ traps, airtight glass container, and titration-based quantification of cumulative CO_2_ and percent mineralization.

The overall processing and testing workflow, from drying and compounding to MFI and biodegradation assays, is summarized in Figure 5.

## 3. Results

### 3.1. Melt-Flow Screening and Matrix Selection

Across neat PLA grades, PTE displayed the highest intrinsic flow (7.00/13.30/24.00 g/10 min at 190/210/230 °C). In PTE blends, BP-1 (PBAT-g-MAH) produced a strong, composition-dependent increase in MFI, whereas BP-2 (PBAT-g-GMA) remained close to neat PLA (Table 1). Balancing nozzle pressure and strand stability for FFF, 10 wt% BP-1 was selected as the compatibilized matrix to incorporate low bamboo loadings (2.5 and 5 wt%). These data establish a printability window for subsequent biodegradation assays and justify focusing the manuscript on BP-1 while retaining BP-2 as screening-only evidence. The lower MFI of the BP-2 systems relative to BP-1 at comparable loadings is consistent with the higher reactivity of GMA, whose epoxy groups can promote chain coupling and light branching, thereby increasing melt viscosity and reducing flow under the D1238 test conditions [7,10,47,48].

**Figure 6:**
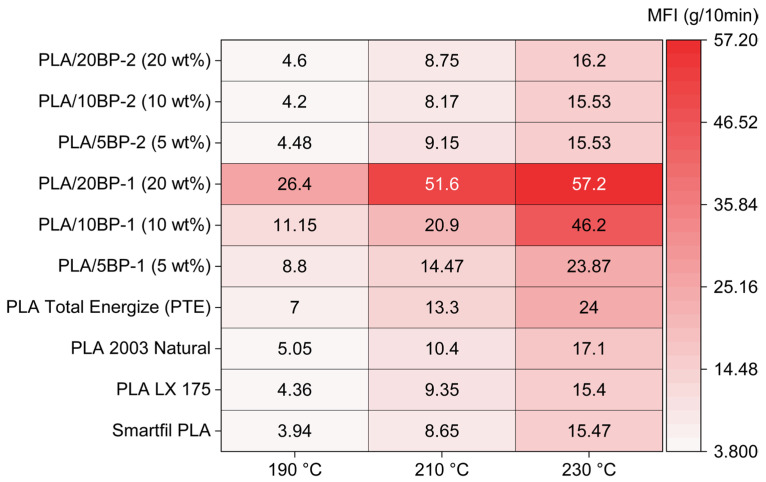
Heatmap of MFI (g/10 min)—Materials x Temperature (ASTM D1238, 2.16 kg).

The increase in MFI with BP-1 is consistent with MAH-based compatibilizers lowering the effective melt viscosity of PTE by improving chain mobility at processing temperatures and reducing elastic recoil during die-swell. In contrast, BP-2 (GMA) can increase the apparent viscosity through epoxy ring-opening reactions that couple or lightly branch the matrix (and react with terminal –OH/–COOH), which keeps MFI close to neat PLA under our conditions. Each condition was measured n = 10 and is reported as mean ± SD in Table 1. The same dataset is also visualized as a heatmap in Figure 6.

These trends align with reports that MAH-based compatibilizers expand the printable window of fiber-filled PLA by improving interfacial stress transfer and reducing porosity/strand rupture during material extrusion [7,10,19,22,47,48,49].

While both PBAT-g-MAH (BP-1) and PBAT-g-GMA (BP-2) were initially evaluated, BP-1 offered the combination of high melt flow and robust filament stability required for FFF and was therefore selected as the compatibilized matrix for all subsequent compounding and biodegradation assays, whereas BP-2 was retained only as MFI screening evidence.

### 3.2. FTIR-ATR Spectra and Reaction Model

**Figure 7:**
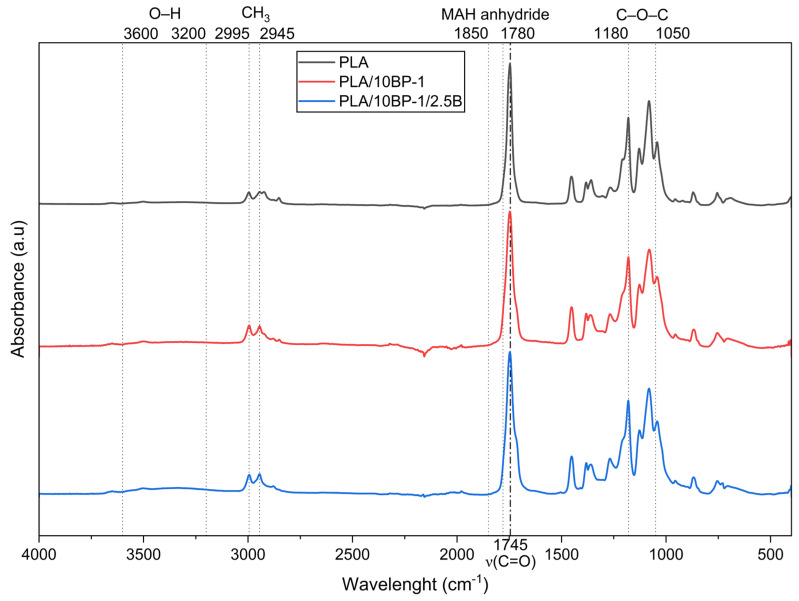
ATR–FTIR spectra of PLA, PLA/10BP-1 and PLA/10BP-1/2.5B (diamond ATR; 4000–600 cm^−1^; 4 cm^−1^; 32 scans). Spectra are baseline-corrected and normalized at 1452 cm^−1^. The ester ν(C=O) at ~1745–1755 cm^−1^ is prominent in all samples; PLA/10BP-1 shows a faint anhydride window at ~1850–1780 cm^−1^, while PLA/10BP-1/2.5B exhibits increased O–H (~3600–3200 cm^−1^) and C–O–C (~1180–1050 cm^−1^) envelopes, consistent with bamboo hydroxyls and polysaccharidic vibrations.

The ATR–FTIR spectra of neat PLA, PLA/10BP-1, and PLA/10BP-1/2.5B (normalized at 1452 cm^−1^) show the expected PLA fingerprint together with subtle, formulation-dependent variations (Figure 7). In all cases, the dominant ester ν(C=O) appears at ~1745–1755 cm^−1^ and the C–O–C stretching bandset is observed within ~1180–1050 cm^−1^; CH_3_ stretching occurs near ~2995–2945 cm^−1^.

Effect of PBAT-g-MAH (BP-1). Relative to neat PLA, PLA/10BP-1 exhibits a slight broadening/intensity increase in the ester ν(C=O) envelope and a discernible shoulder/weak activity within ~1850–1780 cm^−1^, consistent with anhydride-related features from MAH and/or its ring-opened derivatives. Concomitantly, a modest growth of the C–O–C region (~1180–1050 cm^−1^) is observed, compatible with new ester linkages formed after MAH ring opening (esterification with terminal –OH of PLA) and with the PBAT backbone contribution. The absence of a distinct epoxide band at ~910–915 cm^−1^ is consistent with the chemistry of BP-1 (MAH compatibilizer) and helps differentiate it from GMA-based systems.

Such spectral changes are consistent with MAH ring opening followed by esterification with PLA end groups and hydrogen-bonding with lignocellulosic –OH, which jointly improve interfacial cohesion and correlate with the observed processing window (higher MFI) and moderated early-time mineralization. Taken together, these bands support a reaction model in which MAH-grafted PBAT chains undergo ring opening, esterify with PLA end-groups and simultaneously interact via hydrogen bonding with bamboo hydroxyls, as reported for analogous MAH-based compatibilized PLA systems [7,10,47,48].

Effect of bamboo flour loading (2.5 wt%). In PLA/10BP-1/2.5B, the O–H stretching envelope (~3600–3200 cm^−1^) is noticeably enhanced, reflecting the hydroxyl-rich lignocellulosic fraction and/or increased bound moisture; simultaneously, the C–O–C bandset (~1180–1050 cm^−1^) gains intensity compared with PLA/10BP-1, consistent with the contribution of polysaccharidic C–O vibrations from the bamboo flour. The ester carbonyl band remains the most intense feature; small changes in its shape/position relative to neat PLA suggest an altered hydrogen-bonding environment and/or partial ester formation at the interface due to MAH ring opening. Together, these signatures support interfacial compatibilization in the MAH-modified system at low fiber loading, which is coherent with the observed improvements in melt-flow and the stabilized early-time mineralization trends discussed elsewhere in the manuscript.

### 3.3. Early-Time Mineralization at ~21 °C: Carbon Dioxide Produced by the Composite

Net carbon dioxide production (Xₙ), a key indicator of early-time mineralization at 21 ± 2 °C, varied among the three formulations evaluated: PLA (neat), PLA/10BP-1/2.5B and PLA/10BP-1/5B (Table 2). These trends can be explained by the structure and composition of each system.

In the case of PLA (neat), an initial peak of CO_2_ production was observed on day 3 (Xₙ = 2.28), followed by a progressive drop culminating in the lowest value on day 45 (Xₙ = −0.75). This behavior reflects rapid degradation of the amorphous zones of the polymer during the first days, facilitated by their greater accessibility to microbial activity. However, from day 13 onward, the system enters a stabilization phase, probably associated with the resistance of the PLA crystalline domains, whose dense and ordered structure makes their decomposition difficult. This is consistent with previous studies reporting lower biodegradability of neat PLA in the absence of lignocellulosic components [25,27,38,39,40].

In contrast, PLA/10BP-1/2.5B showed a more sustained behavior. Although its initial peak (Xₙ = 2.88 on day 3) was slightly higher than that of PLA (neat), CO_2_ production remained more active in the intermediate stages, with a new local maximum on day 13 (Xₙ = 1.15). This pattern suggests that the presence of 2.5 wt% bamboo powder, together with the BP-1 additive, favors a more efficient interaction between the PLA matrix and the microorganisms. The bamboo fibers begin to decompose after the first few days, providing easily assimilable components such as cellulose and hemicellulose, while the additive acts as a coupling agent, facilitating interfacial compatibility. The pronounced negative value recorded on day 38 (Xₙ = −0.93) likely reflects blank-drift artifacts in a low-signal regime; physically, the preceding and subsequent trends are consistent with a transition toward the degradation of more recalcitrant components such as lignin and the polymeric matrix.

For PLA/10BP-1/5B, which contains 5 wt% bamboo powder, the highest Xₙ value was observed on day 3 (Xₙ = 3.53), followed by a gradual decrease and small oscillations at longer times. This behavior suggests that the additional lignocellulosic fraction provides readily accessible domains for early microbial activity, but that subsequent degradation is constrained by the dense morphology and limited water access under the present mild conditions. Compared with PLA/10BP-1/2.5B, PLA/10BP-1/5B tends to show slightly higher Xₙ values at most time points, but the differences remain modest in magnitude and within the experimental uncertainty of the titrimetric method.

Taken together, the Xₙ profiles indicate that all three materials exhibit very low net mineralization over 0–23 days at ~21 °C. PLA/10BP-1/2.5B and PLA/10BP-1/5B tend to show slightly higher net CO_2_ evolution than PLA (neat) at several time points, but the absolute differences remain small and fall within the experimental uncertainty of the titrimetric method. Under these conditions we interpret the data as evidence of a modest bamboo-driven increase in early-time biodegradation superimposed on a compatibilized, low-porosity morphology that strongly limits overall mineralization. The very low 0–23-day mineralization and the small composite–PLA gap are consistent with ambient studies showing sub-percent CO_2_ evolution at early times and a strong dependence on porosity, compatibilization and fiber chemistry [25,26,27,28,34,36,37,42].

### 3.4. Percent Mineralization of the Mixtures

**Figure 8:**
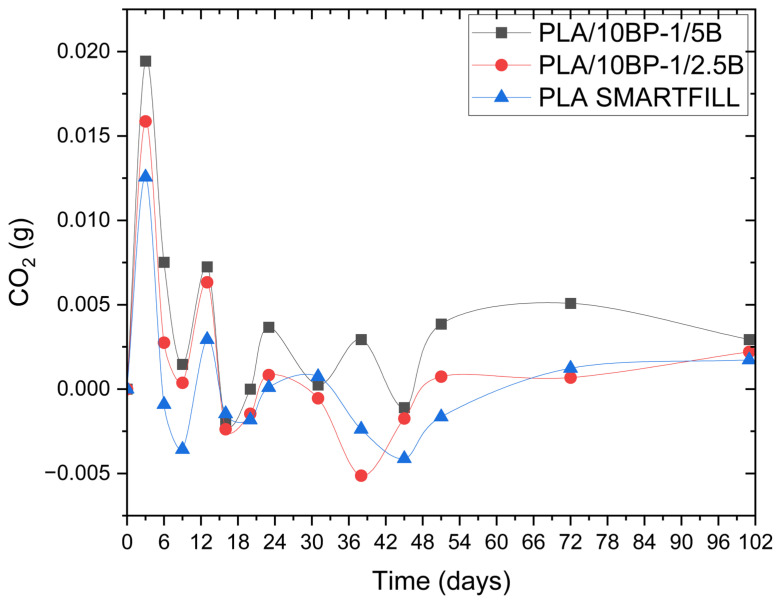
Cumulative CO_2_ generated for PLA (neat), PLA/10BP-1/2.5B and PLA/10BP-1/5B under soil–compost conditions (ASTM D5988; 21 ± 2 °C; n = 3).

**Figure 9:**
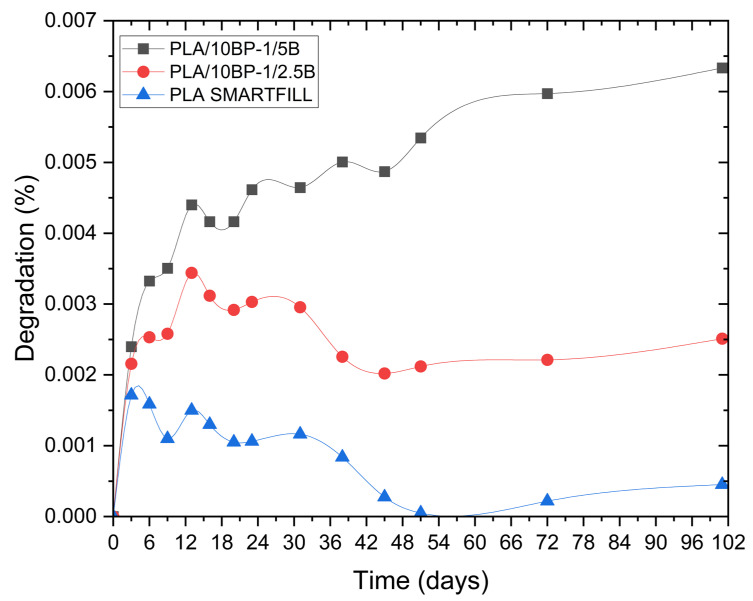
Percent mineralization vs. time for PLA and bamboo-filled composites under soil–compost conditions (ASTM D5988; n = 3).

Figure 8 and Figure 9 illustrate in a comparative way the evolution of percent biodegradation for the three formulations: PLA (neat), PLA/10BP-1/2.5B and PLA/10BP-1/5B. The curves show how bamboo loading and compatibilizer presence modulate the early mineralization response under soil–compost exposure. These graphs allow us to evaluate the kinetics and efficiency of the biodegradation process via the CO_2_ released as a byproduct of the microbial activity on the material.

The incorporation of natural fibers and additives significantly contributed to a slight increase in CO_2_ generation relative to PLA (neat), which represents a direct indicator of the biodegradation process.

In relative terms, PLA/10BP-1/5B exhibited the highest apparent degree of biological decomposition, with slightly higher Xₙ and percent mineralization values than PLA/10BP-1/2.5B at both the initial and final stages of the evaluation period. However, these differences between composites remain modest and fall within the experimental uncertainty of the present setup. PLA (neat), composed exclusively of unreinforced filament, exhibited the lowest overall decomposition response under these mild exposure conditions, with signals close to the titrimetric detection limit. Its curve is characterized by a slight increase in biodegradation during the first days, corresponding to the degradation of more accessible amorphous zones, followed by a prolonged plateau phase, reflecting little subsequent microbial activity. This behavior is consistent with the view that the crystalline structure of pure PLA represents a barrier to biodegradation under natural conditions. From a statistical perspective, the small absolute values of percent mineralization and the n = 3 design mean that formal tests have limited power to discriminate among formulations. Exploratory one-way ANOVA on day 23 percent mineralization, complemented by a Kruskal–Wallis check, did not alter the qualitative reading of the data: differences among PLA (neat), PLA/10BP-1/2.5B and PLA/10BP-1/5B remain modest, and effect-size estimates fall in the “small” range. In line with these constraints, we interpret the ordering PLA/10BP-1/5B ≥ PLA/10BP-1/2.5B ≥ PLA (neat) as a trend toward a bamboo-driven increase in early-time biodegradation, superimposed on a compatibilized, low-porosity morphology that strongly limits the overall extent of mineralization.

## 4. Discussion

### 4.1. Printability Window Set by Melt-Flow Screening

The MFI survey established a clear processing hierarchy among neat PLAs and compatibilized PTE blends. PTE exhibited the highest intrinsic flow among neat grades (190–230 °C), while BP-1 imparted a strong, composition-dependent thinning response to the PTE matrix (up to ~57 g/10 min at 230 °C for 20 wt%), far exceeding BP-2 at comparable loadings. Selecting 10 wt% BP-1 therefore balanced extrudability (viscosity low enough for stable strand deposition) with shape fidelity (viscosity not so low as to promote sag/overfilling), providing a consistent platform to isolate the effect of low bamboo additions (2.5 and 5 wt%) on early biodegradation. This is consistent with prior observations that compatibilizers can expand the printable window of fiber-filled PLA systems by improving interfacial stress transfer and suppressing filament rupture and porosity during FFF/FDM processing [7,10,19,22,30,47,48]. Our selection of 10 wt% BP-1 balances extrudability and shape fidelity, in agreement with optimization studies on natural-fiber/PLA systems for FFF where compatibilizers and parameter tuning jointly govern porosity and bond quality [14,15,16,17,22,49].

### 4.2. Interpreting Negative/Oscillatory Xₙ Segments

Occasional negative increments of the net titration volume (Xₙ) appear in the extended time series. These are a recognized artifact domain when overall CO_2_ evolution is close to the titrimetric detection limit and blanks drift slowly with carbonate uptake; small day-to-day differences in standardization, headspace mixing or water make-up corrections can yield apparent back-titration. The use of triplicates, blank trending and mass-based moisture corrections mitigates bias, and the cumulative series remains monotonic within uncertainty at the level relevant for early-stage comparisons. A compatibilizer-only control (PLA/10BP-1) was not included in the CO_2_ assay because its chemistry and morphology are closer to neat PLA and, at ~21 °C and 0–23 days, it is unlikely to depart measurably from the already sub-percent mineralization regime observed. Instead, we prioritized replicate reactors and blank stability to reduce uncertainty; compatibilizer-only controls will be added in longer and warmer exposures where differences are resolvable. For future work, increasing *n*, tightening blank control (e.g., duplicate traps) and complementing titration with GC-CO_2_ or NDIR would reduce uncertainty bands in the sub-percent mineralization regime and would enable more decisive hypothesis testing on formulation effects.

### 4.3. Practical Implications for FFF/FDM Part Design

From a design-for-use and end-of-life perspective, three consequences are noteworthy:

Processability and quality: The PTE + BP-1 matrix at 10 wt% provides a robust printability window (low nozzle pressure, stable deposition) while retaining adequate melt strength for dimensional accuracy. Low-level bamboo additions (≤5 wt%) do not compromise this window.

Early-life durability: Under ambient service conditions, compatibilized bamboo-filled parts are expected to maintain comparable early surface integrity and property retention to neat PLA, because the observed increase in early mineralization is small in absolute terms. This favors applications that require several weeks to months of functional life before end-of-life routing.

End-of-life routing: The very low 0–23 day mineralization at ≈21 °C underscores that home/ambient soil will not deliver rapid mineralization; industrial composting or thermo-accelerated conditions remain necessary for timely CO_2_ evolution. Designers should communicate realistic disposal guidance and consider disassembly/take-back for printed biocomposite parts.

Our biodegradation objective was to prove how low bamboo loadings behave when embedded in a compatibilized matrix optimized for printing. A PBAT-g-MAH-only control (PLA/10BP-1) would have limited interpretative value here: PBAT-g-MAH is compositionally close to the PLA-based matrix, and without lignocellulosic pathways, its influence on ambient mineralization at ~21 °C is expected to be minor within 23 days. Instead, resources were allocated to replicate reactors and blank stability to reduce uncertainty in the sub-percent mineralization regime. We explicitly frame this as early-time kinetics; future work will include compatibilizer-only controls and temperature sweeps to map long-term effects.

### 4.4. Limitations and Future Work

The present assay intentionally targets early-time kinetics at mild conditions with n = 3. Extending to ≥ 90 days, adding BP-2 comparisons at the same loading, and systematically varying infill/porosity would resolve how processing levers interact with compatibilization and fiber content. Complementary GPC/DSC (molar-mass decay; crystallinity) and µCT (porosity networks) will sharpen mechanism attribution. Finally, using arrhenius-style temperature sweeps (e.g., 21/37/58 °C) can map the inversion point where fibers transition from neutral/retarding to accelerating mineralization.

### 4.5. Significance for 3D Printing

For material-extrusion additive manufacturing, these findings support a materials–process co-design strategy: choose compatibilized matrices with controlled viscosity (here, PTE + BP-1 at 10 wt%) and low fiber loadings to achieve stable printing and strong interlayer bonding, which in turn modulates early biodegradation under ambient exposure. In practice, this means printed bamboo-filled PLA parts can combine (i) easier processing (lower nozzle pressure, fewer defects), (ii) maintained early-life durability in humid environments, despite a modest increase in early mineralization, and (iii) predictable end-of-life when routed to industrial composting, rather than relying on slow ambient mineralization. Such control over both printability and biodegradation onset is directly relevant for consumer goods, fixtures and prototyping where weeks-to-months of service are required before composting pathways are engaged.

## 5. Conclusions

The MFI survey established PTE as the most flowable neat PLA and showed a pronounced thinning effect of BP-1 versus BP-2; PTE + 10 wt% BP-1 provided a robust processing window for FFF with good strand stability and interlayer bonding.

Under near-ambient soil–compost exposure (~21 °C, pH ≈ 7) and within 0–23 days, mineralization was very low for all materials. PLA/10BP-1/2.5B and PLA/10BP-1/5B showed a slight increase in net CO_2_ evolution and percent mineralization relative to neat PLA, but the absolute values remained in the sub-percent regime, consistent with small effect sizes and the qualitative trends identified in the statistical analysis. Interfacial sealing by BP-1 and dense printed infill likely reduced effective water diffusivity, limiting the extent to which the added hydrophilicity of bamboo can accelerate degradation at early times.

The data support a competing-mechanisms view: fibers can increase water access and slightly accelerate early mineralization, but compatibilization and processing that minimize porosity strongly limit the overall extent of degradation at mild temperatures.

For material-extrusion parts intended for weeks-to-months of service, low bamboo loadings with BP-1 compatibilization can improve printability and short-term durability versus neat PLA, while preserving the option of industrial composting for end-of-life; ambient soil alone is insufficient for rapid mineralization.

Extending exposure to ≥ 90 days, adding BP-2 at 10 wt% for parity, and mapping temperature (21/37/58 °C) and infill/porosity will pinpoint the transition where fibers shift from moderating to accelerating degradation; coupling CO_2_ titration with GPC/DSC and μCT will refine mechanism attribution.

## Figures and Tables

**Table 1:** Unified MFI dataset (ASTM D1238, 2.16 kg): neat PLAs and PTE with BP-1/BP-2.

Material	190 °C	210 °C	230 °C
Smartfil PLA (g/10 min)	3.94 ± 0.1	8.65 ± 0.05	15.47 ± 0.12
PLA LX 175 (g/10 min)	4.36 ± 0.25	9.35 ± 0.28	15.40 ± 0.19
PLA 2003 Natural (g/10 min)	5.05 ± 0.12	10.40 ± 0.31	17.10 ± 0.17
PLA Total Energize, PTE (g/10 min)	7.00 ± 0.1	13.30 ± 0.17	24.00 ± 0.4
PLA/5BP-1 (5 wt%)	8.80 ± 0.15	14.47 ± 0.18	23.87 ± 0.35
PLA/10BP-1 (10 wt%)	11.15 ± 0.05	20.90 ± 0.2	46.20 ± 0.5
PLA/20BP-1 (20 wt%)	26.40 ± 0.16	51.60 ± 0.15	57.20 ± 0.16
PLA/5BP-2 (5 wt%)	4.48 ± 0.15	9.15 ± 0.21	15.53 ± 0.23
PLA/10BP-2 (10 wt%)	4.20 ± 0.1	8.17 ± 0.32	15.53 ± 0.18
PLA/20BP-2 (20 wt%)	4.60 ± 0.1	8.75 ± 0.65	16.20 ± 0.15

Note: MFI values in g/10 min. Tests conducted at 190, 210 and 230 °C (ASTM D1238, 2.16 kg). Note: BP-2 (PBAT-g-GMA) used only for MFI screening; biodegradation formulations use BP-1 (PBAT-g-MAH) at 10 wt%.

**Table 2:** Net carbon dioxide production (Xₙ, HCl mL) for PLA (neat), PLA/10BP-1/2.5B and PLA/10BP-1/5B over 101 days of testing under controlled soil–compost conditions.

Day	Xₙ PLA (neat)	Xₙ PLA/10BP-1/2.5B	Xₙ PLA/10BP-1/5B
3	2.2833	2.8833	3.53
6	−0.1667	1.37	0.5
9	−0.65	0.0667	0.27
13	0.5333	1.15	1.32
16	−0.2667	−0.4333	−0.35
20	−0.3333	−0.2667	0
23	0.0167	0.15	0.67
31	0.1333	−0.1	0.04
38	−0.4333	−0.9333	0.53
45	−0.75	−0.3167	−0.2
51	−0.3	0.1333	0.7
72	0.2233	0.1233	0.92
101	0.3133	0.4	0.53

## Data Availability

The original contributions presented in this study are included in the article. Further inquiries can be directed to the corresponding author(s).

## Abbreviations

PLA (polylactic acid); BP-1 (PBAT-g-MAH); BP-2 (PBAT-g-GMA; used only for MFI screening); B (bamboo flour); MFI (melt-flow index); FFF/FDM (fused-filament fabrication); FTIR-ATR (Fourier-transform infrared spectroscopy in attenuated total reflectance). Sample codes used consistently: PLA, PLA/10BP-1, PLA/10BP-1/2.5B, PLA/10BP-1/5B, PLA/10BP-2 (MFI only).
